# The Association of Central Corneal Thickness and Central Corneal Epithelial Thickness with Anthropometric and Biochemical Parameters in Subjects with Impaired Glucose Metabolism

**DOI:** 10.3390/diagnostics15243185

**Published:** 2025-12-13

**Authors:** İhsan Boyacı, Göktuğ Demirci

**Affiliations:** 1 Department of Internal Medicine, Istanbul Medipol University Faculty of Medicine, 34214 Istanbul, Turkey; 2 Department of Ophthalmology, Istanbul Medipol University Faculty of Medicine, 34214 Istanbul, Turkey; gdemirci@medipol.edu.tr

**Keywords:** central corneal thickness, corneal epithelium, impaired glucose metabolism, optical coherence tomography, prediabetes

## Abstract

**Background/Objectives:** Impaired glucose metabolism may alter the corneal structure before overt diabetes develops. This study aimed to assess central corneal thickness (CCT) and central corneal epithelial thickness (CCET) using anterior segment optical coherence tomography (AS-OCT) in individuals with impaired glucose metabolism and to examine their relationships with anthropometric and biochemical parameters. **Methods**: This prospective cross-sectional study included 140 eyes from 70 participants: 20 healthy controls, 17 individuals with insulin resistance, and 33 with prediabetes. CCT and CCET were assessed using AS-OCT. Glucose metabolism was evaluated using a 2 h 75 g oral glucose tolerance test and glycated hemoglobin A1c (HbA1c). Anthropometric measurements, blood pressure, and biochemical parameters were also recorded. **Results**: The mean age of participants was 37.9 ± 12.3 years, and the mean HbA1c was 5.50 ± 0.38%. CCET was significantly higher in the prediabetes group than in the other groups (*p* < 0.01), whereas CCT did not differ significantly. CCET showed significant positive correlations with age, fasting plasma glucose, and HbA1c (all *p* < 0.05). In multivariable linear regression analyses, glycemic parameters remained independently associated with CCET after adjustment for age, sex, and BMI (*p* < 0.05). **Conclusions**: Impaired metabolic processes during prediabetes may influence corneal epithelial thickness. Our findings suggest that corneal parameters obtained by AS-OCT may provide supportive information by highlighting early corneal structural alterations associated with prediabetes. Accordingly, prediabetes detection should not be restricted to HbA1c and OGTT alone. However, longitudinal studies are required before any clinical application can be considered.

## 1. Introduction

Prediabetes is an intermediate state between normal glucose metabolism and overt diabetes and represents a critical period for the early development of metabolic complications. Globally, it currently affects about 373 million individuals, and projections estimate 587 million cases by 2045, highlighting a growing public health burden [1,2]. The risk of progression to type 2 diabetes mellitus (T2D) is as high as 70%, yet the underlying pathophysiological changes during this preclinical stage remain poorly defined [3].

Emerging evidence suggests that microvascular complications may begin during early dysglycemia, even before the onset of overt hyperglycemia. Several studies have reported retinopathy prevalence rates of 6–8% in prediabetic individuals [2,4,5,6], challenging the traditional assumption that such complications occur only after overt hyperglycemia. Diabetic retinopathy (DR) remains a leading cause of vision loss in working-age adults [6]. In T2D, increased corneal stromal thickness and changes in the corneal epithelium have been linked to hyperglycemia and accumulation of advanced glycation end products (AGEs) [7,8,9,10].

Additional metabolic and clinical factors, including hypertension, hyperlipidemia, and disease duration, contribute to microvascular damage [11,12,13,14,15]. Although controlling these risk factors is crucial for preventing retinopathy, predicting its onset or progression remains challenging. Notably, retinopathy may develop even under adequate glycemic control [16]. In contrast, while corneal changes in established T2D have been relatively well characterized, data addressing corneal involvement during the prediabetic stage remain scarce [10].

Neuropathy has also been described in prediabetes, where a reduction in corneal nerve fiber density and length leads to subclinical corneal neuropathy and measurable changes in corneal sensitivity [17,18]. This raises important questions about when DR and other microvascular changes truly begin. Understanding whether retinal and corneal alterations emerge during insulin resistance (IR) and prediabetes is crucial for clarifying the natural history of diabetic microangiopathy and for evaluating potential therapeutic interventions [4,19,20,21].

The cornea, as a metabolically active anterior ocular structure, may serve as a sensitive indicator of early dysglycemia-induced changes. While DR has been extensively characterized, anterior corneal biomarkers —particularly central corneal thickness (CCT) and central corneal epithelial thickness (CCET)—remain understudied in prediabetes. These parameters may reflect early, subclinical epithelial and stromal responses to metabolic dysregulation that occur before clinically overt microvascular complications.

Moreover, potential links between retinopathy and CCT/CCET alterations deserve further exploration, as corneal biomarkers may provide valuable insight into the earliest stages of microvascular dysfunction. Given that up to 40% of individuals with prediabetes progress to diabetes within five years, systematic assessment of corneal parameters using noninvasive imaging techniques such as anterior segment optical coherence tomography (AS-OCT) may facilitate early detection and preventive strategies. Accordingly, the present study aimed to evaluate the impact of impaired glucose metabolism on anterior corneal morphology by quantifying CCT and CCET, and to analyze their associations with key anthropometric and biochemical markers in prediabetic and normoglycemic individuals. We hypothesize that corneal alterations can be detected before the onset of overt diabetes, thereby reflecting early microvascular involvement.

## 2. Materials and Methods

### 2.1. Study Design and Participants

This prospective cross-sectional study included 70 participants (140 eyes) recruited from the internal medicine outpatient clinics at Istanbul Medipol University, Medipol Vatan Health Application and Research Center in 2022. Participants were divided into three groups: 20 healthy controls (HC), 17 insulin-resistant individuals (IR), and 33 with prediabetes. The cohort comprised 42 females and 28 males, aged 21–62 years (Table 1).

Prediabetes was defined according to the American Diabetes Association (ADA) criteria, including fasting plasma glucose (FPG) 100–125 mg/dL (5.6–6.9 mmol/L), 2 h plasma glucose (2 h PG) 140–199 mg/dL (7.8–11.0 mmol/L) on a 75 g oral glucose tolerance test (OGTT), or glycated hemoglobin (HbA1c) 5.7–6.4%. Individuals meeting any of these thresholds but not fulfilling the diagnostic criteria for diabetes were classified as having prediabetes. Insulin resistance (IR) was assessed using the homeostatic model assessment of insulin resistance (HOMA-IR) formula [fasting insulin (µU/mL) × fasting glucose (mg/dL) ÷ 405], with a HOMA-IR ≥ 2.7 indicating IR. Participants demonstrating IR while maintaining normoglycemia were assigned to the IR group, whereas those meeting ADA glycemic thresholds were allocated to the prediabetes group [22].

### 2.2. Exclusion Criteria

Exclusion criteria encompassed individuals with established diabetes mellitus, previous ocular surgery, uveitis, glaucoma, dry eye disease, contact lens use, or recent treatment with growth factors or corticosteroids. Participants who were pregnant, had used systemic antibiotics within the preceding month, or had a history of cardiovascular disease (myocardial infarction, stroke, or peripheral artery disease), chronic systemic inflammatory conditions, or major surgery within the past four weeks were also excluded. Individuals with high refractive error (spherical equivalent > ±3.00 diopters) were excluded a priori to minimize potential confounding effects on corneal epithelial and stromal thickness measurements.

In addition, individuals receiving antihypertensive or lipid-lowering medications were not included because several agents in these drug classes—including beta-blockers, calcium-channel blockers, angiotensin-converting enzyme inhibitors, angiotensin receptor blockers, and statins—may influence corneal thickness, endothelial function, epithelial remodeling, or ocular surface characteristics. These medications could act as pharmacologic confounders, making it difficult to isolate the independent effects of metabolic parameters on CCT and CCET; therefore, participants using these therapies were excluded to preserve internal validity.

### 2.3. Clinical and Anthropometric Assessment

Comprehensive medical history and physical examination were conducted in the outpatient clinic. Body mass index (BMI) and waist-hip ratio (WHR) were assessed during examination. Waist circumference was measured midway between the lower rib edge and iliac crest, while hip circumference was measured at the widest point. WHR values < 0.90 for males and <0.85 for females are considered normal according to criteria defined by the World Health Organization [23]. Neck circumference (NC) was measured below the laryngeal prominence using a tape measure. Body fat percentage (BFP) was assessed using a Tanita MC-780MA body composition analyzer (Tanita Corporation, Tokyo, Japan). Systolic (SBP) and diastolic blood pressure (DBP) were recorded using a manual sphygmomanometer (Erka Perfect Aneroid 201, ERKA, Bad Tölz, Germany). Korotkoff phase I defined systolic pressure, and phase V defined diastolic pressure. Hypertension was defined as SBP ≥ 140 mmHg or DBP ≥ 90 mmHg.

### 2.4. Ophthalmological Examination

Comprehensive ophthalmological examination was included best-corrected visual acuity assessment using Snellen charts under standard illumination. Pre-mydriasis anterior segment evaluation was performed via slit-lamp biomicroscopy (Topcon SL-7F, Topcon Corporation, Tokyo, Japan) to assess corneal pathology, rubeosis iridis, and lens opacification. Following pupillary dilation, vitreous and retinal examination was conducted. Stereoscopic fundoscopy evaluated posterior segment abnormalities including DR, maculopathy, vascular changes, optic neuropathy, and chorioretinal scarring. Fundus photography and fluorescein angiography were performed when indicated. AS-OCT was subsequently acquired.

### 2.5. AS-OCT Measurements

Optical coherence tomography provides non-contact, high-resolution cross-sectional imaging for intraocular structure evaluation. Anterior segment and corneal assessment was performed using the Revo FC system (software version 11.0.4; Optopol Technology, Zawiercie, Poland), a multipurpose device enabling both anterior and posterior segment imaging. The topography module generates axial, epithelial, and CCT maps using 850 nm SLD technology with 80,000 measurements per second and 2.4 mm scan depth in standard mode [24]. Built-in software automatically produces 8 mm-diameter epithelial maps [25].

To ensure measurement reliability, all AS-OCT scans were obtained following a standardized acquisition protocol. Patients were instructed to fixate on the internal target to maintain centration, and images with motion artifacts or poor centration were discarded. The device’s built-in quality assessment software was used to confirm adequate signal strength, and only scans meeting the manufacturer’s recommended minimum quality threshold were included. When necessary, scans were repeated to achieve optimal imaging quality. All measurements were performed by a single experienced ophthalmologist to eliminate inter-operator variability and enhance reproducibility. Both eyes of each participant were included in the analysis, provided that image quality criteria were met for each eye; thus, a total of 140 eyes were evaluated. To minimize the potential effect of diurnal variations on CCT and CCET measurements, all AS-OCT examinations were performed during the same time window, between 09:00 and 12:00 AM.

### 2.6. Rationale for Biochemical and Anthropometric Parameters

The biochemical and anthropometric parameters included in this study were selected based on their established relevance to metabolic dysfunction, which is known to influence corneal structure in individuals with impaired glucose metabolism. Hyperglycemia and elevated HbA1c have been linked to corneal epithelial alterations through inflammatory and glycation-related pathways [10]. Anthropometric indices such as BMI, WHR, BFP, and NC reflect overall and regional adiposity, which are strongly associated with metabolic burden and may plausibly influence CCT and CCET. Therefore, these variables were selected to represent key metabolic factors with potential effects on corneal morphology.

### 2.7. Laboratory Analysis

Blood and urine samples were obtained after ≥10 h of overnight fasting. Venous blood was collected in 8.5 mL serum separator tubes (Becton Dickinson, Franklin Lakes, NJ, USA), and HbA1c samples were drawn into 2 mL EDTA tubes. All specimens were stored at −80 °C until analysis. HbA1c was measured using boronate affinity chromatography (Quo-Lab^®^, EKF Diagnostics PLC, Cardiff, UK) and expressed as % according to the National Glycohemoglobin Standardization Program (NGSP).

Plasma glucose and FPG were measured by the hexokinase method on a Roche Cobas Integra^®^ 400 Plus analyzer using the GLUC kit (Roche Diagnostics, Mannheim, Germany). Impaired fasting glucose (IFG) and/or impaired glucose tolerance (IGT) were assigned a standard 2 h 75 g OGTT.

C-peptide was measured to provide additional validation of pancreatic β-cell function and insulin secretion capacity, complementing HOMA-IR calculations. Unlike insulin, C-peptide is not metabolized by the liver and provides a more stable indicator of endogenous insulin production. This dual assessment (HOMA-IR and C-peptide) allows for a more comprehensive evaluation of insulin resistance and β-cell function in the progression from normal glucose tolerance to prediabetes. Fasting insulin and C-peptide were measured on a Roche Cobas e601 analyzer using the Elecsys Insulin and Elecsys C-peptide assays with electrochemiluminescence immunoassay (ECLIA) methodology. Insulin resistance was calculated using the HOMA-IR index, as previously described.

Total cholesterol (T-col), triglycerides (TG), high-density lipoprotein cholesterol (HDL-c), and low-density lipoprotein cholesterol (LDL-c) were measured on a Roche Cobas Integra^®^ 400 Plus analyzer using the CHOL2, TRIGL, HDLC4, and LDL-C3 kits, respectively, with enzymatic colorimetric methods (Roche Diagnostics, Mannheim, Germany). Urinary albumin was measured on a Roche Cobas Integra^®^ 400 Plus analyzer using the ALBT2 kit with an immunoturbidimetric method (Roche Diagnostics, Mannheim, Germany). The estimated glomerular filtration rate (GFR) was calculated using the Chronic Kidney Disease Epidemiology Collaboration (CKD-EPI) equation.

### 2.8. Statistical Analysis

Descriptive statistics were presented as numbers and percentages for categorical variables, and as mean ± standard deviation or median (minimum–maximum) for numerical variables. For comparisons among independent groups, the Kruskal–Wallis test was used for non-normally distributed data, and one-way ANOVA for normally distributed variables. Post hoc comparisons were performed using Tukey, Dunnett’s, or Tamhane’s T2 tests based on variance homogeneity. The chi-square (χ^2^) test was used to compare categorical variables. Correlation analyses were conducted using Pearson’s or Spearman’s correlation coefficients, as appropriate. To further justify the choice of statistical procedures, data distribution characteristics were evaluated using the Shapiro–Wilk test, which is recommended for modest sample sizes. Accordingly, parametric or non-parametric tests were selected based on the distributional properties of each variable. To control for type I error inflation associated with multiple comparisons, Bonferroni-adjusted significance thresholds were applied when necessary. Only adjusted *p*-values were interpreted as statistically significant in the presence of multiple testing. A *p*-value < 0.05 was otherwise considered statistically significant.

Additionally, to examine the independent associations of biochemical and anthropometric variables with corneal thickness parameters, multivariable linear regression analyses were performed using central corneal thickness right (CCTR), central corneal thickness left (CCTL), central corneal epithelial thickness right (CCETR), and central corneal epithelial thickness left (CCETL) as dependent variables in separate models. Age, sex, BMI, and relevant biochemical markers were included as covariates. Variables were selected based on their significance in univariate analyses and clinical relevance. Unstandardized regression coefficients (β) and 95% confidence intervals were reported. Effect sizes for between-group comparisons were calculated using eta-squared (η^2^) for overall group differences and Cohen’s d for pairwise analyses. All analyses were conducted using SPSS v22.0 (IBM Corp., Armonk, NY, USA).

## 3. Results

The study included 70 participants (140 eyes): 20 (28.5%) in the HC group, 17 (24.3%) in the IR group, and 33 (47.2%) in the prediabetes group. Demographic and biochemical characteristics including age, sex distribution, HbA1c, FPG, and anthropometric parameters are summarized in Table 1. CCTR was 535 ± 35 µm, CCTL was 530 ± 53 µm, CCETR was 60.8 ± 5.7 µm, and CCETL was 61.0 ± 5.7 µm.

Normality testing demonstrated that several variables (including 2 h PG, IR, blood pressure, NC, LDL-c, TG, albuminuria, ALT, CCTL, CCETR, and CCETL) did not follow a normal distribution, whereas BMI, WHR, BFP, HbA1c, fasting insulin, and CCTR were normally distributed (Table 1).

The prediabetes group had a significantly higher mean age and elevated anthropometric indices (BMI, WHR, NC, and BFP) compared with the HC and IR groups (Table 2 and Table 3).

Significant negative correlations were identified between BMI and CCTL, as well as between BFP and bilateral CCT (*p* < 0.05). Conversely, CCET demonstrated a significant positive correlation with NC (*p* < 0.05) (Table 4).

SBP was significantly higher in the prediabetes group compared with the other two groups (*p* < 0.05) (Table 2). A significant negative correlation was also observed between CCTL and SBP (*p* < 0.05) (Table 4).

Biochemical parameters including FPG, TG, LDL-c, and ALT were significantly higher in the prediabetes group (all *p* < 0.01). Additionally, HbA1c, fasting insulin, fasting C-peptide, T-col, and eGFR differed significantly among groups (*p* < 0.01) (Table 2). Regarding corneal measurements, CCTL was significantly higher in the control group (*p* < 0.01), whereas both CCETR and CCETL were significantly higher in the prediabetes group (*p* < 0.01). To account for potential confounding, a multivariable linear regression model with CCTR as the dependent variable and age, sex, BMI, and biochemical parameters as covariates was constructed; none of these variables remained an independent predictor of CCTR after adjustment (Supplementary Table S1).

Spot creatinuria was significantly higher in the prediabetes group (*p* < 0.05) (Table 2 and Table 3). In correlation analyses, both CCETR and CCETL demonstrated significant positive associations with age, HbA1c, and FPG (CCETR: *p* < 0.001; CCETL: *p* < 0.05). CCETR was also positively correlated with T-col, TG, and LDL-c (*p* < 0.05), whereas CCETL showed a positive correlation with TG and a negative correlation with HDL-c (*p* < 0.05). Additionally, FPG demonstrated a significant negative correlation with bilateral CCT (*p* < 0.05) (Table 4). To further account for potential confounding, a separate multivariable linear regression model was constructed with CCTL as the dependent variable and age, sex, BMI, and biochemical parameters as covariates; none of these variables remained an independent predictor of CCTL after adjustment (Supplementary Table S2). Given the observed associations between metabolic parameters and epithelial thickness, an additional multivariable linear regression model was constructed with CCETR as the dependent variable and age, sex, BMI, and biochemical parameters as covariates; HbA1c remained an independent predictor of CCETR after adjustment (Supplementary Table S3). Similarly, HbA1c, age, FPG, and TG were identified as independent predictors of CCETL after adjustment (Supplementary Table S4). Effect sizes for overall and pairwise between-group differences in key corneal and metabolic parameters are presented in Supplementary Table S5.

## 4. Discussion

Several studies have reported that diabetic retinopathy may already emerge during the prediabetic stage, with a prevalence of 6–8% [2,4,5,6]. Because the corneal epithelium is avascular, it obtains glucose from the aqueous humor via the transcorneal route rather than directly from the bloodstream. In diabetes, hyperglycemia-induced inflammatory and oxidative stress pathways have been associated with corneal epithelial damage, delayed wound healing, and an increased risk of neurotrophic keratitis. Moreover, increased CCT and the resulting relative corneal stiffness may lead to falsely elevated intraocular pressure measurements [10,26,27,28].

The corneal epithelium serves as a protective barrier and an integral component of the innate immune defense of the eye. Disruption of this layer increases susceptibility to infection and impairs refractive function, while diabetic keratopathy is characterized by defective epithelial repair and compromised barrier integrity [29,30]. In healthy individuals, CCT is approximately 540–560 μm and may increase by 10–30 μm in patients with T2D, proportional to disease duration [8,9,10,27,28]. The corneal epithelium is a stratified, non-keratinized layer measuring approximately 48–53 μm and exhibits regional asymmetry in healthy eyes [31,32]. In well-controlled patients with T2D without retinopathy, increased CET has been reported, suggesting that this finding may be considered an early indicator of ocular involvement [33].

In our dataset, mean CCTR and CCTL values in individuals with prediabetes were 529 ± 36 µm and 520 ± 67 µm, respectively. CCET has been reported to be approximately 50 µm in healthy individuals [10]. In the present study, mean CCETL (60.5 ± 4.6 µm) and CCETR (59.8 ± 4.5 µm) were significantly higher than those observed in the other two groups, suggesting that CCET may begin to increase during the prediabetic stage.

Although the observed CCET differences were small and measured at the micron level, they are unlikely to independently affect routine clinical decision-making. Rather, they should be interpreted as indicators of early, subclinical corneal remodeling associated with impaired glucose metabolism. Such microstructural changes may become relevant in longitudinal follow-up or in settings sensitive to corneal microarchitecture, while CCET cannot currently be considered a stand-alone diagnostic or prognostic marker. Longitudinal studies are therefore required to clarify whether these epithelial alterations progress or gain clinical significance. Notably, limited comparable evidence exists in prediabetes, with only one study reporting reduced macular thickness at the retinal level [19].

Individuals with prediabetes in our cohort were older than those in the healthy control and insulin-resistance groups, consistent with previous reports [4]. Given the association between age and CCET, age-adjusted multivariable analyses were performed and confirmed that metabolic parameters particularly HbA1c and FPG remained independently associated with CCET. As diabetes duration has been linked to increased CCT in type 2 diabetes, the potential impact of early metabolic impairment on corneal structure during the prediabetic stage warrants further investigation [24,34].

Conversely, the greater CCT observed in the HC group compared with individuals with prediabetes may indicate that corneal thickening initially occurs at the epithelial level, which could explain the higher CCET observed in prediabetes. Alternatively, in healthy controls, the corneal stroma may contribute to optimal refraction by regulating electrolyte and water exchange with the endothelium [10,34,35,36].

Beyond disease duration, hyperglycemia and the accumulation of AGEs may contribute to corneal structural alterations [9,26]. Hyperglycemia-related oxidative stress has been shown to impair Na^+^/K^+^-ATPase activity, potentially promoting basement membrane thickening [10,26,27,36], while higher HbA1c levels in prediabetes have been associated with increased DR risk [4]. In our cohort, elevated glycemic, lipemic, and insulin-related parameters in prediabetic individuals paralleled the observed increase in CCET, supporting the contribution of early metabolic dysregulation to corneal epithelial thickening prior to overt diabetes.

Higher C-peptide levels observed in the prediabetes group may indicate compensatory hyperinsulinemia, reflecting β-cell attempts to overcome IR and supporting our HOMA-IR findings. Hyperglycemia may further impair epithelial cell growth, migration, and adhesion by altering growth factor signaling pathways [30,37], thereby increasing susceptibility to epithelial injury and delayed repair [9].

CCET demonstrated significant positive correlations with age, FPG, HbA1c, T-col, TG, and LDL-c. Together, these findings suggest that corneal epithelial alterations emerge as part of a broader metabolic continuum, preceding the clinical diagnosis of diabetes and paralleling systemic metabolic deterioration [10]. Future multicenter studies with larger sample sizes are required to confirm these preliminary findings and improve generalizability.

Prediabetic individuals exhibited a higher BFP than healthy controls. Notably, BFP was negatively correlated with bilateral CCT, whereas no significant association was observed with CCET. This pattern suggests that increased adiposity may differentially affect corneal stromal properties rather than epithelial thickness. Obesity-associated metabolic alterations, including chronic low-grade inflammation and altered collagen metabolism, may contribute to these stromal changes [38,39,40]. Previous population-based studies have reported inconsistent findings regarding the association between obesity and CCT, with some showing no relationship [38,39] and others identifying significant associations with body weight, BMI, FPG, and 2-h PG levels [40]. In our study, obesity-related anthropometric measures were elevated in individuals with prediabetes and demonstrated distinct, layer-specific associations with corneal parameters: central adiposity indices such as WHR and NC correlated positively with CCET, whereas BMI and BFP were inversely associated with CCT. These findings suggest that obesity-related metabolic burden may differentially affect corneal epithelial and stromal compartments, potentially through interactions with glycemic and lipemic dysregulation in prediabetes.

CCT and CCET exhibited opposite correlation patterns with obesity- and glycemia-related parameters, suggesting layer-specific corneal responses to early metabolic dysregulation rather than a true paradox. These divergent patterns may reflect alterations in stromal hydration, endothelial pump function, and extracellular matrix remodeling described in early diabetic keratopathy [10]. To account for the interplay among metabolic variables, multivariable regression analyses were performed and confirmed that glycemic parameters remained independently associated with CCET after adjustment for age, sex, and BMI. Although endothelial and biomechanical assessments were not performed, these findings support the concept that epithelial changes may precede stromal remodeling during early metabolic impairment, consistent with prior AS-OCT studies in T2D [8,9,10,27,28,33].

Most patients with T2D present to ophthalmology clinics after clinically evident DR has developed, underscoring the importance of early detection at the prediabetic stage. Current guidelines recommend comprehensive ophthalmologic evaluation at the time of T2D diagnosis and at regular intervals thereafter, while no specific screening recommendations exist for individuals with prediabetes or obesity [41,42,43]. Although ocular research in diabetes has largely focused on the retina, the cornea remains relatively underexplored, particularly in prediabetic populations. In this context, and given the cross-sectional design and sample size, the present findings should be interpreted as preliminary and hypothesis-generating rather than as evidence supporting routine ophthalmologic screening.

The divergent epithelial and stromal thickness patterns observed in our study likely reflect layer-specific corneal responses to early metabolic dysfunction rather than a true paradox. Increased CCET in prediabetes and obesity may be related to low-grade inflammation and dysglycemia-associated epithelial alterations, whereas reduced stromal CCT may reflect inflammation-driven extracellular matrix remodeling and obesity-related metabolic effects [10]. These compartment-dependent responses suggest that the cornea undergoes early structural remodeling during the transition from normoglycemia to IR and prediabetes, consistent with prior reports linking adiposity markers to CCT changes [38,39,40].

This study should be interpreted in light of several methodological constraints. The cohort showed an unbalanced group distribution with a relatively small IR subgroup and an overall modest sample size, which may limit between-group comparisons. The single-center, cross-sectional design and absence of neuropathy assessment further restrict causal inference. Accordingly, these findings should be considered preliminary and hypothesis-generating, pending confirmation in longitudinal studies with serial AS-OCT assessments.

Hyperglycemia and hypertension remain the most important modifiable risk factors for preventing DR progression and vision loss [12]. Although strict glycemic control and HbA1c monitoring before overt diabetes have been emphasized, HbA1c alone may be insufficient for early risk stratification [4]. These considerations highlight the potential value of a multifactorial approach incorporating OGTT alongside clinical and metabolic risk factors, as current ADA recommendations rely primarily on HbA1c-based screening and may not fully capture early metabolic dysregulation [43,44]. In parallel, biomarker-based strategies and improved interdisciplinary access may help refine early risk assessment. In this context, integrated digital health approaches combining anthropometric measures, non-invasive ocular parameters, and metabolic biomarkers should be regarded as exploratory and hypothesis-generating avenues for future investigation.

Beyond individual associations, integrating corneal metrics with anthropometric and metabolic markers may inform the development of multifactorial risk models for early ocular alterations in prediabetes. However, such approaches remain exploratory and require validation in larger, longitudinal cohorts.

## 5. Conclusions

Metabolic dysregulation appears to begin during the prediabetic stage and may be associated with early corneal epithelial alterations, as reflected by increased CCET. These findings suggest that corneal involvement may represent an early ocular manifestation along the metabolic disease continuum. However, longitudinal studies are required to determine whether such epithelial changes represent the earliest stage of a progressive metabolic trajectory.

Although AS-OCT may provide a non-invasive means of detecting subtle anterior segment changes in prediabetes, its clinical use should currently be regarded as exploratory and adjunctive rather than as a screening tool. Finally, early corneal epithelial alterations may represent a potentially modifiable target, warranting further investigation into interventions addressing epithelial regeneration and oxidative stress during prediabetes.

## Figures and Tables

**Table 1:** Basic characteristics of the participants enrolled in study.

Variables	Units	Healthy Control	Insulin Resistance	Prediabetes	All Participants	*p*-Value
Number	-	20	17	33	70	
Female sex	n (%)	16 (80.0)	12 (70.6)	14 (42.4)	-	
Age	year	28.1 ± 8	30.5 ± 9	47.6 ± 7	37.9 ± 12	0.002 *
BMI	kg/m^2^	21.7 ± 2.8	25.4 ± 4.6	29.1 ± 3.8	26.1 ± 4.8	0.200
WHR	cm	0.78 ± 0.12	0.82 ± 0.09	0.90 ± 0.06	0.84 ± 0.10	0.200
NC	cm	34.7 ± 2.3	37.5 ± 4.2	42.5 ± 3.3	39.1 ± 4.7	0.025 *
BFP	%	22.2 ± 6.6	27.3 ± 6.2	26.9 ± 6.8	25.7 ± 6.9	0.200
SBP	mmHg	108 ± 7	117 ± 5	119 ± 8	116 ± 8	0.001 *
DBP	mmHg	77 ± 4	88 ± 1	80 ± 2	79 ± 3	0.001 *
HbA1c	%	5.25 ± 0.21	5.25 ± 0.30	5.78 ± 0.31	5.5 ± 0.38	0.200
FPG	mmol/L	5.13 ± 0.35	5.38 ± 0.36	5.87 ± 0.55	5.52 ± 0.56	0.056
2 h PG	mmol/L	6.61 ± 0.10	6.17 ± 0.65	6.60 ± 1.69	6.54 ± 1.57	0.005 *
FI	µU/mL	8.13 ± 3.90	17.07 ± 4.60	13.82 ± 6.61	12.9 ± 6.30	0.200
IR	-	1.80 ± 0.90	4.05 ± 1.1	3.87 ± 2.7	3.34 ± 2.2	0.009 *
FC-p	ng/mL	1.8 ± 0.5	2.8 ± 0.6	2.7 ± 0.8	2.5 ± 0.8	0.200
T-col	mmol/L	4.29 ± 0.87	4.76 ± 0.34	5.52 ± 1.08	4.93 ± 1.09	0.200
TG	mmol/L	0.74 ± 0.20	1.29 ± 0.20	1.44 ± 0.50	1.20 ± 0.60	0.001 *
LDL-c	mmol/L	2.41 ± 0.80	2.82 ± 0.70	3.52 ± 1.04	3.04 ± 0.10	0.017 *
HDL-c	mmol/L	1.50 ± 0.30	1.33 ± 0.30	1.33 ± 0.30	1.39 ± 0.30	0.200
Albuminuria	mg	9.07 ± 2.10	7.66 ± 4.30	6.21 ± 4.70	7.30 ± 7.30	0.001 *
Creatinuria	mg	115 ± 75	143 ± 79	173 ± 76	149 ± 79	0.200
GFR	mL/min/1.73 m^2^	112 ± 13	116 ± 14	100 ± 11	111 ± 16	0.200
ALT	IU/L	13 ± 9	14 ± 7	25 ± 17	19 ± 14	0.001 *
CCTR	μm	550 ± 33	528 ± 29	529 ± 36	535 ± 35	0.082
CCTL	μm	550 ± 32	528 ± 29	520 ± 67	530 ± 53	0.001 *
CCETR	μm	59.8 ± 4.5	57.1 ± 4.6	63.1 ± 5.9	60.8 ± 5.7	0.029 *
CCETL	μm	60.5 ± 4.4	57.3 ± 5.1	63 ± 5.9	61 ± 5.7	0.028 *

BMI: Body mass index; WHR: Waist/hip ratio; NC: Neck circumference; BFP: Body fat percentage; SBP: Systolic blood pressure; DBP: Diastolic blood pressure; HbA1c: Glycated haemoglobin; FPG: Fasting plasma glucose; 2 h PG: 2 h plasma glucose; FI: Fasting insulin; IR: Insulin resistance; FC-p: Fasting C-peptide; T-col: Total cholesterol; TG: Triglyceride; ALT: Alanine aminotransferase; LDL-c: Low-density lipoprotein cholesterol; HDL-c: High-density lipoprotein cholesterol; GFR: Glomerular filtration rate; CCTR: Central corneal thickness (right); CCTL: Central corneal thickness (left). Overall sex distribution: 42 females and 28 males. Continuous variables are expressed as mean ± standard deviation. (*) Indicates statistically significant differences between groups (*p* < 0.05). *p*-values for continuous variables were calculated using one-way ANOVA or Kruskal–Wallis tests, as appropriate based on data distribution. No *p*-value was calculated for sex distribution.

**Table 2:** The relationship of non-normally distributed variables according to the groups (mean ranks).

Variables	Units	Healthy Control	Insulin Resistance	Prediabetes	*p* ^a^-Value	*p* ^b^-Value	*p* ^c^-Value
Age	year	20.03	23.12	51.26	0.691	0.001 *	0.001 *
NC	cm	15.84	27.81	48.18	0.037 *	0.001 *	0.001 *
SBP	mmHg	18.35	38.59	44.30	0.001 *	0.001 *	0.133
DBP	mmHg	28.65	37	38.88	0.057	0.017 *	0.459
2 h PG	mmol/L	26	19	19.97	0.770	0.084	0.846
IR	-	17.28	48.35	39.92	0.001 *	0.001 *	0.201
TG	mmol/L	19.65	34.53	43.77	0.052	0.001 *	0.186
LDL-c	mmol/L	21.83	31.13	44.86	0.166	0.001 *	0.018 *
Albuminuria	mg	35.29	36.94	28.68	0.122	0.001 *	0.167
ALT	IU/L	23.68	28.34	45.67	0.044 *	0.001 *	0.003 *
CCTL	μm	44.43	31.41	31.03	0.030 *	0.045 *	0.823
CCETR	μm	31.20	21.81	43.70	0.071	0.001 *	0.001 *
CCETL	μm	32.98	21.44	42.80	0.024 *	0.002 *	0.001 *

NC: Neck circumference; SBP: Systolic blood pressure; DBP: Diastolic blood pressure; 2 h PG: 2 h plasma glucose; IR: Insulin resistance; TG: Triglyceride; LDL-c: Low-density lipoprotein cholesterol; ALT: Alanine aminotransferase; CCTL: Central corneal thickness (left); CCETR: Central corneal epithelial thickness (right); CCETL: Central corneal epithelial thickness (left). Values are presented as mean ranks. *p* ^a^: Comparison between Group 1 and Group 2; *p* ^b^: Comparison between Group 1 and Group 3; *p* ^c^: Comparison between Group 2 and Group 3. (*) Indicates statistically significant differences between groups (*p* < 0.05). Overall *p*-values were calculated using the Kruskal–Wallis test, followed by pairwise post hoc comparisons. Only variables with non-normal distribution are presented in this table.

**Table 3:** The relationship of the normally distributed variables according to the groups.

Variables	Units	Healthy Control	Insulin Resistance	Prediabetes	*p*-Value
BMI	kg/m^2^	16.1–25.9	18.2–33.2	19.6–36.6	0.001 *
WHR	cm	0.65–1.17	0.65–0.98	0.71–1.02	0.001 *
BFP	%	10.1–30.8	18.7–42.0	15.2–39.1	0.034 *
HbA1c	%	4.80–5.60	4.57–5.60	4.84–6.30	0.001 *
FPG	mmol/L	4.44–5.55	4.72–6.06	4.61–6.94	0.001 *
FI	μU/mL	3.27–22.11	11.37–24.39	2.68–31.80	0.001 *
FC-p	ng/mL	1.12–3.32	1.93–4.18	1.00–4.60	0.001 *
T-col	mmol/L	2.69–5.28	3.70–6.52	3.98–9.34	0.001 *
HDL-c	mmol/L	0.80–2.04	0.72–1.99	0.85–2.40	0.130
Creatinuria	mg	8–309	29–260	38–379	0.001 *
GFR	mL/min/1.73 m^2^	90–138	90–136	76–120	0.001 *
CCTR	μm	508–635	491–595	475–616	0.074

BMI: Body mass index; WHR: Waist/hip ratio; BFP: Body fat percentage; HbA1c: Glycated haemoglobin; FPG: Fasting plasma glucose; FI: Fasting insulin; FC-p: Fasting C-peptide; T-col: Total cholesterol; HDL-c: High-density lipoprotein cholesterol; GFR: Glomerular filtration rate; CCTR: Central corneal thickness (right). Values are expressed as minimum–maximum. (*) Indicates statistically significant differences between groups (*p* < 0.05). *p* values were calculated using the One-Way ANOVA test.

**Table 4:** Correlation analysis of corneal parameters and demographic, anthropoemetric and biochemical data.

Variables	Units	CCTR (μm)		CCTL (μm)		CCETR (μm)		CCETL (μm)	
		r	*p*	r	*p*	r	*p*	r	*p*
Age	year	−0.056	−0.649	−0.052	0.670	0.367	0.002 *	0.293	0.014 *
BMI	kg/m^2^	−0.224	0.067	−0.245	0.044 *	0.210	0.085	0.141	0.250
WHR	cm	0.018	0.882	0.006	0.962	0.253	0.037 *	0.223	0.068
NC	cm	−0.086	0.486	−0.106	0.391	0.252	0.038 *	0.277	0.022 *
BFP	(%)	−0.306	0.011 *	−0.342	0.004 *	0.010	0.934	−0.013	0.917
SBP	mmHg	−0.234	0.053	−0.250	0.038 *	0.088	0.474	0.013	0.918
DBP	mmHg	−0.172	0.157	−0.177	0.146	0.232	0.055	0.228	0.059
HbA1c	%	−0.220	0.069	−0.221	0.069	0.441	0.001 *	0.334	0.005 *
FPG	mmol/L	−0.253	0.036 *	−0.269	0.025 *	0.371	0.002 *	0.271	0.024 *
2 h PG	mmol/L	−0.189	0.248	−0.209	0.202	−0.175	0.285	−0.048	0.772
FI	μU/mL	−0.150	0.219	−0.184	0.130	−0.138	0.259	−0.161	0.187
IR	-	−0.161	0.186	−0.200	0.099	−0.059	0.629	−0.100	0.412
FC-p	ng/mL	−0.170	0.165	−0.198	0.105	0.010	0.914	0.029	0.811
T-col	mmol/L	−0.052	0.677	−0.077	0.536	0.254	0.038 *	0.201	0.102
TG	mmol/L	−0.087	0.484	−0.092	0.461	0.288	0.018 *	0.317	0.009 *
LDL-c	mmol/L	−0.056	0.648	−0.082	0.508	0.241	0.048 *	0.172	0.160
HDL-c	mmol/L	−0.121	0.330	−0.119	0.337	−0.221	0.073	−0.259	0.035 *
Albuminuria	mg	−0.140	0.275	−0.163	0.201	−0.070	0.585	−0.125	0.330
Creatinuria	mg	0.065	0.605	0.067	0.595	0.209	0.095	0.184	0.142
GFR	mL/min/1.73 m^2^	0.076	0.550	0.079	0.537	−0.267 *	0.033	−0.270	0.031 *
ALT	IU/L	0.021	0.865	−0.004	0.973	0.153	0.214	0.118	0.337

BMI: Body mass index; WHR: Waist/hip ratio; NC: Neck circumference; BFP: Body fat percentage; SBP: Systolic blood pressure; DBP: Diastolic blood pressure; HbA1c: Glycated haemoglobin; FPG: Fasting plasma glucose; 2 h PG: 2 h plasma glucose; FI: Fasting insulin; IR: Insulin resistance; FC-p: Fasting C-peptide; T-col: Total cholesterol; TG: Triglyceride; LDL-c: Low-density lipoprotein cholesterol; HDL-c: High-density lipoprotein cholesterol; GFR: Glomerular filtration rate; CCTR: Central corneal thickness (right); CCTL: Central corneal thickness (left); CCETR: Central corneal epithelial thickness (right); CCETL: Central corneal epithelial thickness (left); (*) Indicates statistically significant correlations at *p* < 0.05. Correlation coefficients (r) and *p* values were calculated using Spearman’s rank correlation analysis.

## Data Availability

All data supporting the reported results are included within the article. For additional information, inquiries can be directed to the corresponding author.

## Supplementary Materials

The following supporting information can be downloaded at: https://www.mdpi.com/article/10.3390/diagnostics15243185/s1.

## Abbreviations

ADA	American Diabetes Association
AGEs	Advanced Glycation End Products
ALT	Alanine Transaminase
AS-OCT	Anterior Segment-Optical Coherence Tomography
BFP	Body Fat Percentage
BMI	Body Mass Index
CCET	Central Corneal Epithelial Thickness
CCETL	Central Corneal Epithelial Thickness Left
CCETR	Central Corneal Epithelial Thickness Right
CCT	Central Corneal Thickness
CCTL	Central Corneal Thickness Left
CCTR	Central Corneal Thickness Right
CKD-EPI	Chronic Kidney Disease Epidemiology Collaboration
DBP	Diastolic Blood Pressure
DR	Diabetic Retinopathy
FPG	Fasting Plasma Glucose
GFR	Glomerular Filtration Rate
HC	Healthy Controls
HDL-c	High-Density Lipoprotein Cholesterol
HOMA-IR	Homeostatic Model of Assessment—Insulin Resistance
IFG	Impaired Fasting Glucose
IGT	Impaired Glucose Tolerance
IR	Insulin Resistance
LDL-c	Low-Density Lipoprotein Cholesterol
NC	Neck Circumference
NGSP	National Glycohemoglobin Standardization Program
OGTT	Oral Glucose Tolerance Test
SBP	Systolic Blood Pressure
TG	Triglyceride
T-col	Total Cholesterol
T2D	Type 2 Diabetes Mellitus
WHR	Waist/Hip Ratio
2 h PG	2 hour Plasma Glucose
