# Supplementary material for: The Association of Central Corneal Thickness and Central Corneal Epithelial Thickness with Anthropometric and Biochemical Parameters in Subjects with Impaired Glucose Metabolism
Source: Diagnostics (Basel). 2025 Dec 13;15(24):3185. doi: 10.3390/diagnostics15243185 (PMC12732270; doi:10.3390/diagnostics15243185)
Supplement: Supplementary file 1 [file diagnostics-15-03185-s001.zip › diagnostics-3901240-supplementary.pdf]

Supplementary Material

Title: The Association of Central Corneal Thickness and Central Corneal Epithelial Thickness with Anthropometric and Biochemical Parameters in Subjects with Impaired Glucose Metabolism

Supplementary Table S1. Multivariable linear regression analysis identifying independent predictors of central corneal thickness (CCTR)

Predictor	Units	β (unstandardized)	95% CI for β	p-value
Intercept	–	682.88	513.12 to 852.65	–
Age	year	0.25	–0.91 to 1.42	0.665
Sex (male)	–	8.53	–12.73 to 29.79	0.425
BMI	kg/m^2	–1.77	–4.15 to 0.60	0.141
FPG	mmol/L	–0.94	–2.10 to 0.23	0.112
HbA1c	%	1.27	–32.18 to 34.73	0.940
T-col	mmol/L	2.71	–3.51 to 8.92	0.387
TG	mmol/L	–0.51	–1.73 to 0.71	0.409
LDL-c	mmol/L	–2.70	–8.90 to 3.49	0.386
HDL-c	mmol/L	–3.32	–9.57 to 2.92	0.291

CCTR: Central corneal thickness (right); BMI: Body mass index; HbA1c: Glycated haemoglobin; FPG: Fasting plasma glucose; T-col: Total cholesterol; TG: Triglyceride; LDL-c: Low-density lipoprotein cholesterol; HDL-c: High-density lipoprotein cholesterol. β values represent unstandardized regression coefficients. CI indicates confidence interval. p values < 0.05 were considered statistically significant.

Supplementary Table S2. Multivariable linear regression analyses identifying independent predictors of central corneal thickness (CCTL)

Predictor	Units	β (unstandardized)	95% CI for β	p-value
Intercept	–	682.88	513.12 to 852.65	–
Age	year	−0.31	−0.84 to 0.21	0.245
Sex	–	−2.74	−9.12 to 3.64	0.398
BMI	kg/m^2	−0.42	−1.08 to 0.24	0.209
HbA1c	%	−1.58	−6.92 to 3.76	0.560
FPG	mmol/L	−1.21	−3.02 to 0.61	0.187
TG	mmol/L	−0.33	−1.91 to 1.26	0.683
LDL-c	mmol/L	−0.28	−2.15 to 1.59	0.767
HDL-c	mmol/L	0.74	−1.46 to 2.95	0.506

CCTL: Central corneal thickness (left); BMI: Body mass index; HbA1c: Glycated haemoglobin; FPG: Fasting plasma glucose; T-col: Total cholesterol; TG: Triglyceride; LDL-c: Low-density lipoprotein cholesterol; HDL-c: High-density lipoprotein cholesterol. β values represent unstandardized regression coefficients. CI indicates confidence interval. p values < 0.05 were considered statistically significant.

Supplementary Table S3. Multivariable linear regression analyses identifying independent predictors of central corneal epithelial thickness (CCETR)

Predictor	Units	β (unstandardized)	95% CI for β	p-value
Intercept	–	42.18	21.31 to 63.05	–
Age	year	0.05	−0.01 to 0.11	0.094
Sex (male)	–	−0.88	−2.75 to 0.99	0.356
BMI	kg/m^2	0.07	−0.10 to 0.24	0.417
FPG	mmol/L	0.21	−0.12 to 0.54	0.212
HbA1c	**%**	2.84	0.91 to 4.77	0.004*
T-col	mmol/L	0.18	−0.41 to 0.77	0.551
TG	mmol/L	0.29	−0.06 to 0.64	0.105
LDL-c	mmol/L	0.37	−0.21 to 0.95	0.207
HDL-c	mmol/L	−0.42	−1.09 to 0.25	0.217

CCETR: Central corneal epithelial thickness (right); BMI: Body mass index; HbA1c: Glycated haemoglobin; FPG: Fasting plasma glucose; T-col: Total cholesterol; TG: Triglyceride; LDL-c: Low-density lipoprotein cholesterol; HDL-c: High-density lipoprotein cholesterol. β values represent unstandardized regression coefficients. CI indicates confidence interval. p values < 0.05 were considered statistically significant.

Supplementary Table S4. Multivariable linear regression analysis identifying independent predictors of central corneal epithelial thickness left (CCETL)

Predictor	Units	β (unstandardized)	95% CI for β	p-value
Intercept	-	43.01	22.14 to 63.88	-
Age	year	0.06	0.01 to 0.12	0.021*
Sex (male)	-	-0.74	-2.59 to 1.11	0.430
BMI	kg/m2	0.03	-0.13 to 0.19	0.701
FPG	mmol/L	0.24	0.01 to 0.47	0.039*
HbA1c	%	2.12	0.36 to 3.88	0.019*
T-col	mmol/L	0.15	-0.39 to 0.69	0.585
TG	mmol/L	0.31	0.05 to 0.57	0.021*
LDL-c	mmol/L	0.19	-0.29 to 0.67	0.440
HDL-c	mmol/L	-0.48	-1.07 to 0.11	0.107

CCETL: Central corneal epithelial thickness (left); BMI: Body mass index; HbA1c: Glycated haemoglobin; FPG: Fasting plasma glucose; T-col: Total cholesterol; TG: Triglyceride; LDL-c: Low-density lipoprotein cholesterol; HDL-c: High-density lipoprotein cholesterol. β values represent unstandardized regression coefficients. CI indicates confidence interval. p values < 0.05 were considered statistically significant.

Supplementary Table S5. Effect sizes for between-group comparisons of corneal and metabolic parameters

Variable	Comparison	Effect size metric	Value	Interpretation
CCETR	Overall (3 groups)	η^2	0.19	Large
CCETL	Overall (3 groups)	η^2	0.16	Large
CCTR	Overall (3 groups)	η^2	0.08	Moderate
CCTL	Overall (3 groups)	η^2	0.06	Moderate
HbA1c	Overall (3 groups)	η^2	0.47	Very large
CCETR	Prediabetes vs Control	Cohen's d	0.61	Moderate–large
CCETL	Prediabetes vs Control	Cohen's d	0.47	Moderate
CCETR	Prediabetes vs Insulin resistance	Cohen's d	1.09	Very large
CCETL	Prediabetes vs Insulin resistance	Cohen's d	1.00	Very large

CCETR, central corneal epithelial thickness (right eye); CCETL, central corneal epithelial thickness (left eye); CCTR, central corneal thickness (right); CCTL, central corneal thickness (left); HbA1c, glycated hemoglobin A1c.
Effect sizes were calculated using eta-squared (η^2) for overall group comparisons and Cohen's d for pairwise comparisons.
Values of $\eta^2 \geq 0.14$ and Cohen's $d \geq 0.8$ were considered indicative of large effects.
